# Lasalocid immediately and completely prevents the myocardial damage caused by coronary ischemia reperfusion in rat heart

**DOI:** 10.1007/s11010-018-3437-2

**Published:** 2018-09-06

**Authors:** Sergio F. Estrada-Orihuela, Carlos Ibarra-Pérez

**Affiliations:** 1 Centro de Invención e Innovación Tecnológica de México, S.C., Dakota 14-8, Col. Nápoles, C. P. 03810, Ciudad de México, Deleg. Benito Juárez México; 2 Retorno de Los Leones 58, C. P. 01710, Ciudad de México, Mexico

**Keywords:** Heart, Myocardial infarct, Coronary reperfusion, Myocardial ischemia, Lasalocid

## Abstract

Lasalocid, a specific mobile membrane ionophore for calcium, dopamine and norepinephrine was assayed in its capacity to reduce or maintain unaltered the cardiovascular function in conditions of imminent myocardial injury. In experiments of coronary blockade and reperfusion carried out in rat heart, it was found that when administered from 5 to 30 minutes prior to the induction of coronary blockade, at a concentration of 2 mg/kg of body weight, the ionophore immediately, simultaneously, and completely interrupts the blood pressure decay, cardiac frequency increase, electrical ventricular tachycardia and fibrillation, as well as the fall of mitochondrial oxidative phosphorylation and decay of mitochondrial oxygen uptake provoked by the induced myocardial injury. It appears that the molecular mode of action of the lasalocid is associated with its unique ability to transport both calcium and the catecholamines, dopamine and norepinephrine, across mitochondrial and bimolecular lipid membranes, as well as through synaptic cell membrane terminals from rat heart, myocardial fibers of the heart and heart chromaffin membrane vesicles. It is suggested that for the potential medical use of lasalocid to detain incoming ischemic myocardial damage, there exists a need to develop a personal electronic device able to simultaneously monitor, detect, and inform on the very early and simultaneous signs of cardiac alterations of electrical, mechano-chemical, metabolic and hydraulic nature, all which precede heart failure and to administer the lasalocid.

## Introduction

Lasalocid is a molecule with the chemical structure of a polyether, formerly known as compound HLR-X537A, which carries out a dual function, both as an antibiotic and as a mobile carrier of calcium and catecholamine (dopamine and norepinephrine). In its function as an antibiotic, lasalocid is very useful in the prevention, treatment and control of a variety of animal infectious and parasitic diseases [1]. It promotes the growth and weight increase in livestock [2] and elevates the efficiency of feed utilization in poultry [3] and swine [4]. Lasalocid has no significant negative collateral effects [1–4], particularly at the low concentrations assayed and from the single dose administered in this study.

As demonstrated in this document, lasalocid also has a unique capacity to control and maintain unaltered the cardiovascular function in conditions of imminent myocardial injury.

Our laboratory was the first to show that lasalocid transports calcium through mitochondrial membranes [5] as well as through artificial bimolecular lipid membranes [6], forming stereo-specific complexes with Ca^2+^, in nuclear magnetic resonance studies carried out in phosphatidyl choline liposomes [7]. Stempel and Westley isolated this molecule from actinomycetes [8] and were the first to show [9] that lasalocid increased the contractile force of the heart and its inotropic properties, without affecting the heart rhythm or its cardiac frequency. Other research groups have shown that lasalocid transports the catecholamines dopamine and norepinephrine through artificial bimolecular lipid bilayers [10], as well as through isolated rat heart membrane chromaffin vesicles [11], myocardial fibers of hamster heart, unrelated to external calcium [12], and synaptosomal membranes from rat heart [13]. The above presumably occurring through an exchange diffusion mechanism involving the free unprotonated carbonyl anionic group of lasalocid [10, 11].

After a number of years passed from the publication of the above studies, and the most relevant scientific contributions apparently had been published on the lasalocid effects in cardiomyocytes, the following findings by Brooks et al. [14], Stamm et al. [15], and Xie et al. [16] showing the massive accumulation and overload of calcium occurring in myocardial muscle fibers during the blockage and reperfusion of rat coronary artery, which precedes the installment of induced sudden myocardial damage, came to our attention. Taking this into account, as well as the more recent data on lasalocid, cited in forthcoming pages, we decided to embark and explore the possibility that lasalocid could diminish or prevent the sudden myocardial damage caused by blockage and reperfusion of rat coronary artery.

The results from this work clearly indicate that indeed lasalocid, when administered from 5 to 30 min prior to the induction of myocardial injury, at one single dose and, at a concentration of 2 mg/kg body weight, immediately and totally interrupts the sudden myocardial damage and anoxia caused by blockage and reperfusion of coronary artery in laboratory rats, thereby maintaining the mitochondrial energy conserving properties of the cardiomyocyte to carry out oxidative phosphorylation and coupled oxygen uptake.

## Methods

The methodology described by Manning and Hearse [17] Chávez et al. [18], and Parra et al. [19] was used in these experiments as follows: (*n*) = 20 male Wistar rats, weighing between 280 and 300 g, were anesthetized with sodium pentobarbital (60 mg/kg, i. p), and maintained under assisted respiration, by means of tracheotomy. Respiratory frequency was maintained at 72 ventilations per minute. Lasalocid was administered i. v. (*n* = 20) at a concentration of 10 µM, in 100 µl, ethanol-dimethyl formamide 3:1, through the femoral vein, 10 min before the start of the experiment. The time and dose were chosen, after evaluating in the present experimental contribution a dose–response relationship in vivo, at concentrations of 2 × 10^− 5^; 6 × 10^− 5^; 10 × 10^− 5^; 14 × 10^− 5^ and 18 × 10^− 5^M, where lasalocid efficiently transports dopamine and norepinephrine, [11–13], as well, as Ca^+ 2^ through biological [5] and bimolecular lipid membranes [6, 10]. In dose–response experiments, three rats were evaluated, in identical experimental conditions, at each indicated concentrations. The replicates, within any single experiment, were averaged, to obtain a single value for each experimental series.

The untreated group (*n* = 20), only received NaCl solution (0.9%), with 100 µl, of 3:1 ethanol-dimethyl formamide, the adjuvant where lasalocid was dissolved. The chest was opened by left thoracotomy, the left coronary artery was isolated near its origin, by an intramural 6.0 silk loop. The occlusion of the artery was attained by passing a short tube over the vessel and clamping it firmly with the thread. The ischemic stage lasted 5 min, as established by Manning and Hearse [17], Chávez et al. [18], and Parra et al. [19]. Removing the clamp started reperfusion. Heart rate and electrocardiographic variations were monitored by means of three surface electrodes, placed in standard D II position. Blood pressure was measured with a pressure transducer attached to a femoral cannula.

In separate experiments, mitochondrial oxygen uptake and oxidative phosphorylation were measured, with the oxidizable substrates, glutamate plus malate or β hydroxybutyrate, in control and reperfused hearts, with or without lasalocid. Mitochondria were isolated, by homogenizing in 250 mM mannitol, 70 mM sucrose, and 1 mM EDTA. Two subsequent washes and final suspension of the mitochondria were done in the sample medium without EDTA. Oxygen uptake was measured by means of vibrating platinum electrode. Respiration and phosphorylation were measured, as in (Ferguson and Estrada-O et al. [20], in the conditions, of Table 1.

**Table 1:** Respiratory control and oxidative phosphorylation of heart mitochondria, isolated from ischemic control hearts (*n* = 10) and from ischemic hearts treated (*n* = 10) with lasalocid

Experimental conditions ΔQO_2_ (*N*) represents the change in microliters of O2 per mg of nitrogen	Glutamate + malate		β hydroxybutyrate	
	ΔQO2 (*N*)	P: 0 ratio	ΔQO2 (*N*)	P: 0 ratio
Control (no reperfusion)	242	2.8 ± 0.12	168	1.8 ± 0.15
Reperfusion of myocardium	160	1.6 ± 0.03	76	0.62 ± 0.11
Reperfusion plus lasalocid	238	2.5 ± 0.12	154	1.65 ± 0.18

The reaction mixture of each experimental condition, contained: 2 mM ATP: 13 mM phosphate- TEA buffer, Ph 7.4; 3 mM Mg Cl2; 15 mM KCl; 140 mM sucrose and 2.2 Mg of mitrogen, in 0.5 mM sucrose. Hexokinase and 50 µmol of glucose were added at zero time. The oxidizable substrate β hydroxybutyrate was added to given concentration of 10 mM. When l-malate was present with glutamate, it was 5 mM. Lasalocid was administered 5 min before ischemia reperfusion, final volume 3 ml: temperature, 30 °C. ΔQO2 (*N*) represents the change in microliters of O2 per mg of nitrogen. Protein was measured by the biuret procedure. Oxidative phosphorylation was assayed with β hydroxybutyrate as oxidizable substrate in five rats from ischemic control hearts and five experimental animals from ischemic hearts treated with lasalocid. Same as with glutamate plus malate as oxidizable substrate. Obtained experimental values between the two groups were statistically significant (*p* 0.001). Differences in oxygen uptake obtained with glutamate plus malate, as well as β hydroxybutyrate in control and reperfused hearts, with or without lasalocid, were statistically significant (*p* 0.001)

All animal procedures were performed according with the Guide for the Care and Use of Laboratory Animals, published by The National Institutes of Health. (No. 85 − 23, revised 1985, as well as in compliance with the standing National Mexican Norm Specifications for the Production, Care and Use of Laboratory Animals (NOM-062-ZOO-1999). It is to be noted that the investigations were carried out and initiated over three years ago at the National Institute of Cardiology of Mexico, as well as at the Metropolitan University of Mexico, whereby such investigations received the verbal clearance and support of collaborating scientists from both institutions to conduct the studies. However, after duly complying with the above standards, a document to be granted by the respective Institutional Ethics Committee of such institutions was not requested at the beginning of the experiments, concerning the proper Care and Use of Laboratory Animals.

## Results

**Fig. 1:**
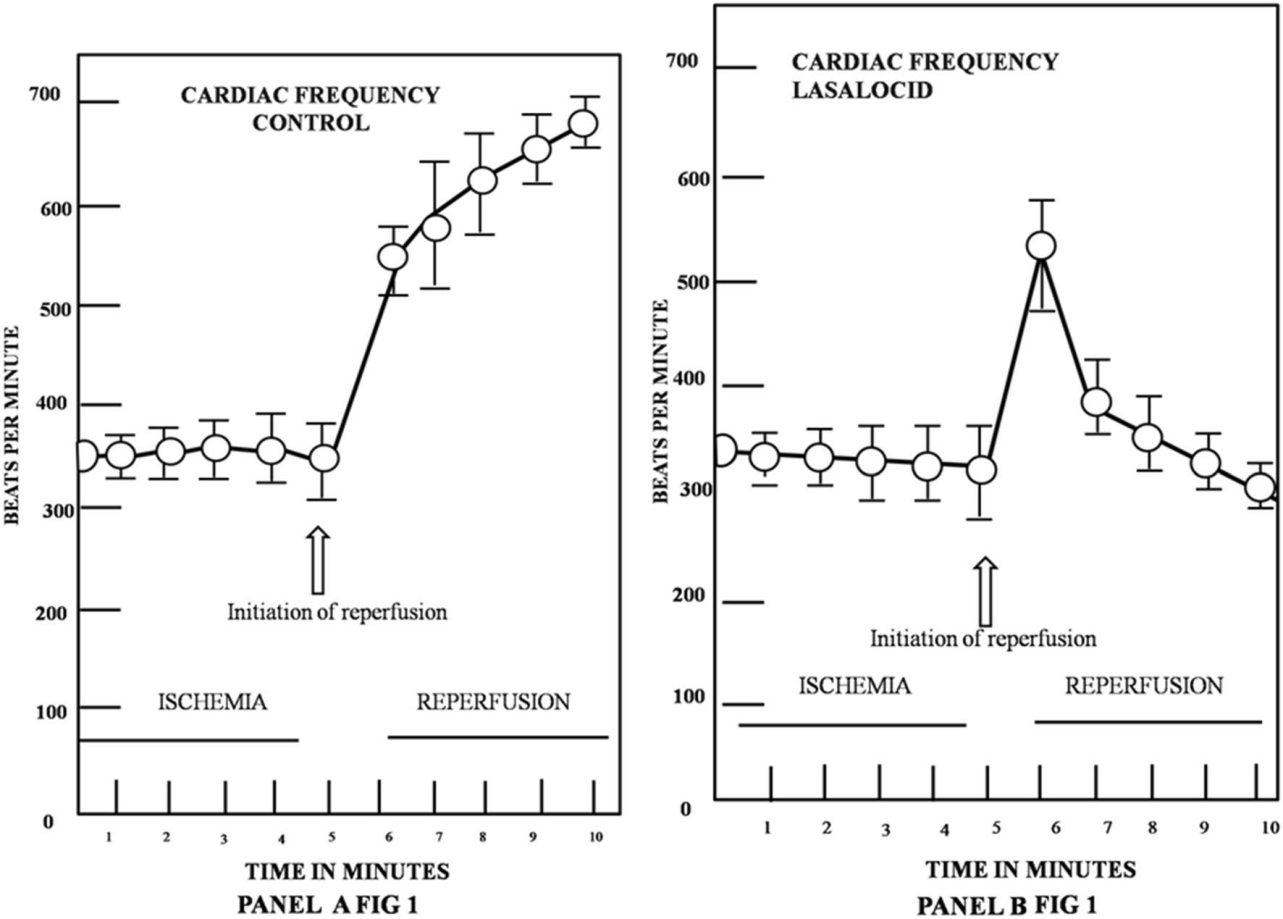
**a** Effect of coronary blockage reperfusion in the cardiac frequency of five control rats. Who received 0.9% saline solution, containing 100 µl of ethanol-dimethyl formamide 3:1, instead of lasalocid, 5 min before the blockage reperfusion. Coronary blockage was carried out at zero time of the graph and reperfusion was executed 5 min after the blockage. Values are expressed as mean ± SEM. **b** Effect of coronary blockage and reperfusion in the cardiac frequency of 5 rats, which received 100 µl of dimethyl formamide ethanol 3:1, containing 10 µM lasalocid. Injected intravenously in the femoral vein, 5 min before the blockage reperfusion. Coronary blockage was carried out at zero time of the graph and reperfusion was executed 5 more minutes after the blockage. Values are expressed as mean ± SEM

As shown in the statistical analysis of data described in Panel A of Fig. 1, in control rats which received 0.9% saline solution, added with 100 µl of adjuvant to solvate the ionophore (ethanol-dimethyl-formamide 3:1), there were no significant changes in cardiac frequency during the 5-min period that followed coronary blockage (closed circles). However, 1 min immediately after blood reperfusion was initiated following coronary blockage, the cardiac frequency was considerably increased from the normal value of 350, to 600–700 beats per min. After this period, cardiac frequency in untreated rats remained at high values and lasted from 5 to 15 more minutes, whereby nearly 40–50% death of experimental subjects occurred in this period. In only less than 10% of animals, cardiac frequency went down 10–15 min after reperfusion. On the other hand, as shown in the statistical analysis of data described in Panel B of Fig. 1, in all rats (*n* = 20) that received 100 µl, of 10 µM lasalocid, from 5 to 30 min before the induction of coronary blockage and reperfusion, cardiac frequency remained unaltered during the 5-min period that followed coronary blockage (open circles). However, in this group of experimental animals exposed to lasalocid, contrary to the results observed in control rats, coronary reperfusion provoked a 1-min rapid, cyclic and transient increase from 350 to 500–550 beats per minute in cardiac frequency. This was followed, exactly 1 min after, by an immediate return and recovery of heart frequency to normal and stable values of 300–350 beats per minute that was maintained normal and stable, even 1 h or more, after the start of the experiment.

**Fig. 2:**
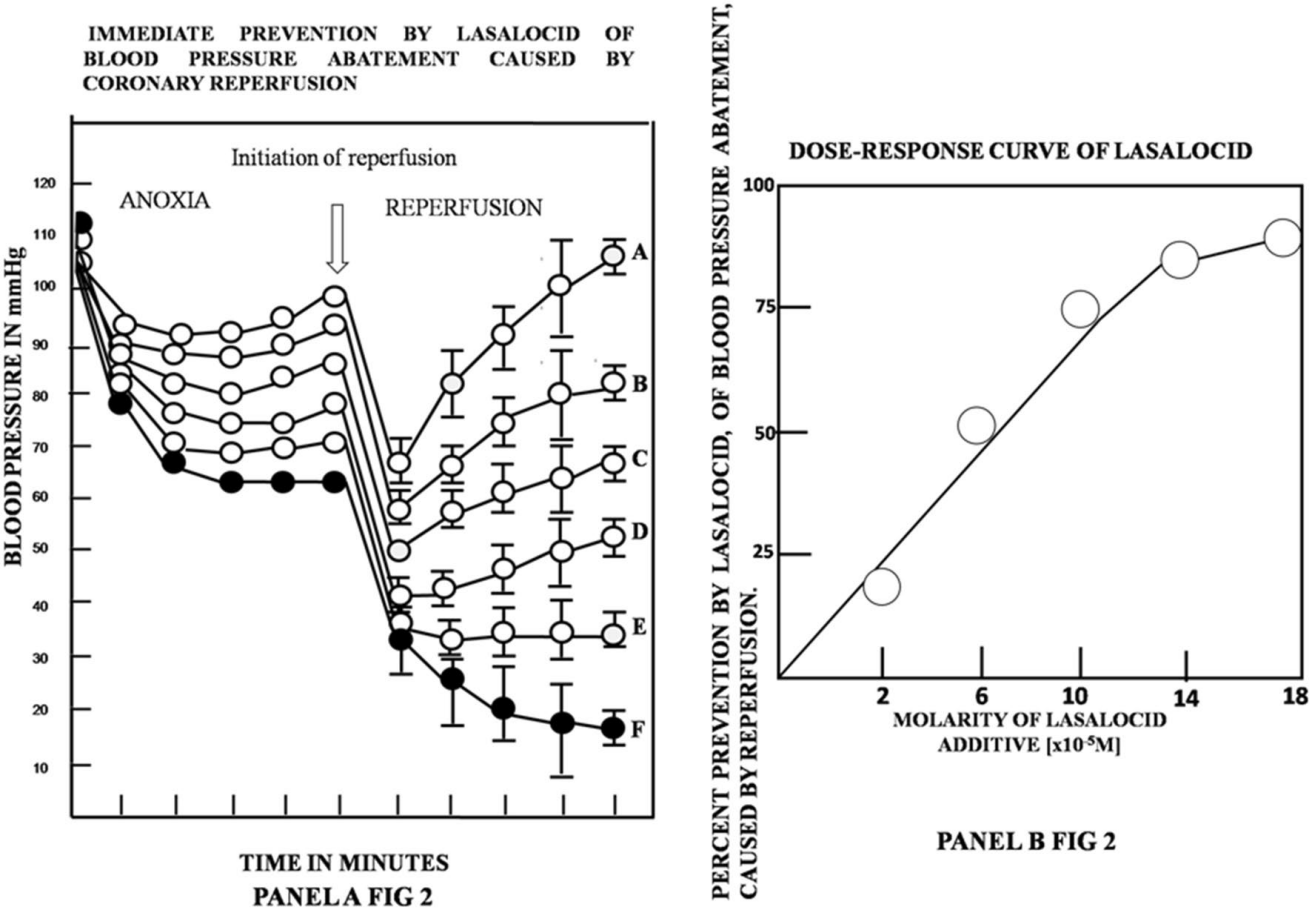
Dose–response experiments, studying five different concentrations of lasalocid, in triplicate assays for each individual dose, for evaluating the immediate prevention and restoration, by lasalocid, of the blood pressure abatement, caused by coronary blockade and reperfusion. Trace described in closed circles was obtained in control rats, receiving 0.9% saline solution, and 100 µl of adjuvant ethanol: dimethyl formamide instead of lasalocid, 5 min before the blockage reperfusion. Coronary blockage was carried out at zero time of the graph and reperfusion was executed 5 min after the blockage. The five different traces described in open circles from (**a**) represent the blood pressure values in mM Hg obtained at the indicated moment of the induced coronary blockage and reperfusion and at the different concentrations of lasalocid tested. Trace F, described in closed circles correspond to blood pressure values obtained in untreated rats without lasalocid. Trace from curve A was obtained at a concentration of lasalocid of 18 × 10(− 5) M; curve B was obtained at the concentration of lasalocid of 14 × 10(− 5) M; curve C was obtained at the concentration of lasalocid of 10 × 10(− 5) M; curve D was obtained at the concentration of lasalocid of 6 × 10(− 5) M; and curve E was obtained at the concentration of lasalocid of 2 × 10(− 5) M. Three rats were evaluated, in identical experimental conditions at each of the five indicated concentrations of lasalocid described in open circles. The replicates within any single experiment were averaged to obtain a single value for each experimental series. Other conditions of each individual experiment were the same, as described in Panel B of Fig. 1. Values are expressed as mean ± SEM

The statistical analysis of Panels A and B of Fig. 2 shows a clear-cut dose–response to immediately, gradually and efficiently detain the blood pressure abatement caused by coronary blockade and reperfusion, at five different concentrations of lasalocid (open circles). Panel A of Fig. 2 depicts, in untreated rats (closed circles), that during the 5-min period that followed coronary blockage, the blood pressure steadily decreased from 110 to 120 mmHg, to approximately 65–60 mm Hg. Nonetheless, 1 min immediately after coronary reperfusion was induced, blood pressure abruptly decreased from 60 to 20 mm Hg, during the following 5–15 min. It is also apparent in the statistical analysis of data described in Panel B of Fig. 2 that, in the relationship between the additive molarity of lasalocid and the percentage of prevention of blood pressure abatement by the drug, the ionophore action is directly dependent on the translocator concentration. Moreover, in a mirror image of Panel B of Fig. 1, Panel A of Fig. 2 shows that the ionophore, in only seconds, not only prevents, but also fully and completely restores and reverses to normal values the decay of blood pressure. This condition was maintained normal 1 h and beyond the initiation of the experiment.

**Fig. 3:**
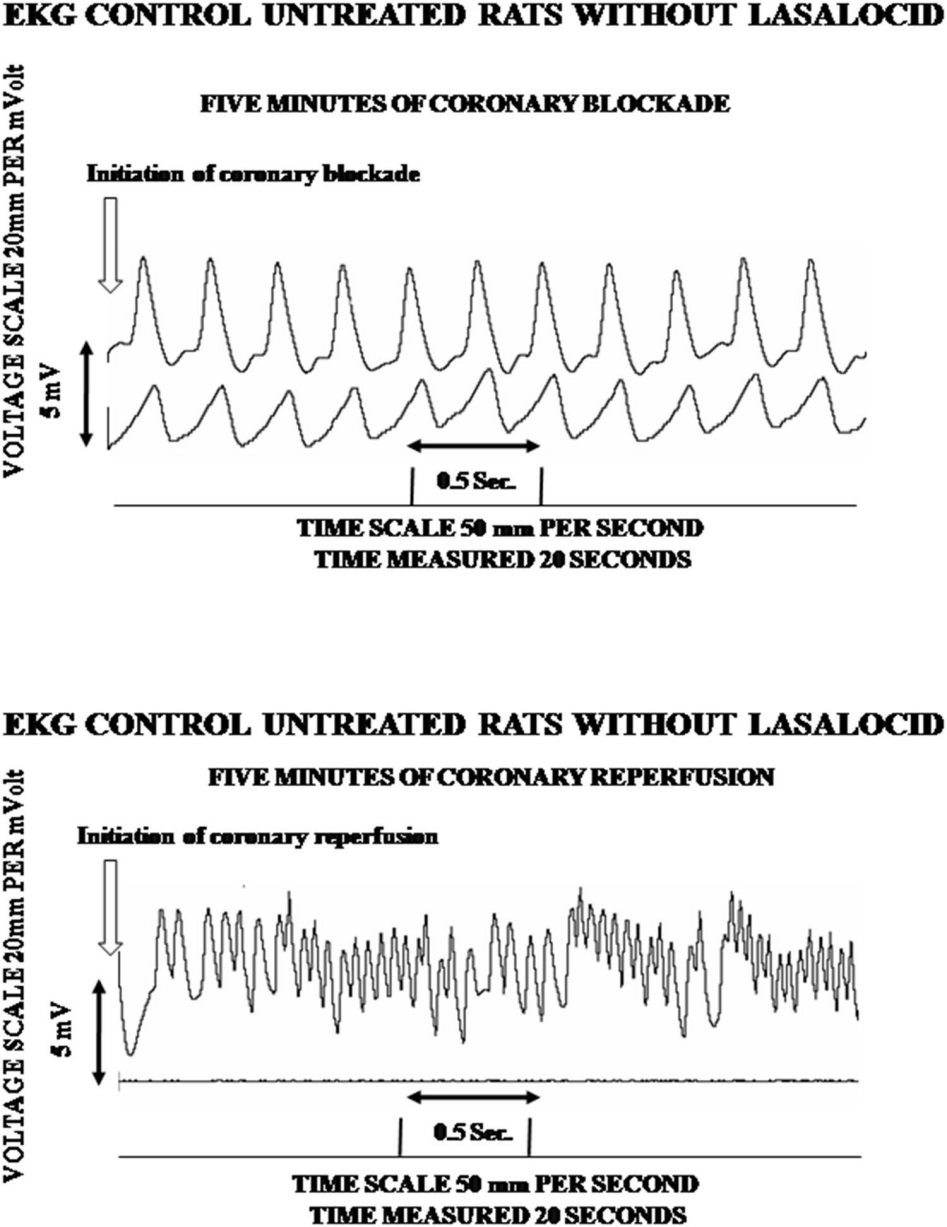
Effect of coronary blockage and reperfusion in the typical electrocardiogram of 5 control rats who received 0.9% saline solution, and 100 µlt of adjuvant of ethanol: dimethyl formamide 3:1 instead of lasalocid, 5 min before the blockage reperfusion. Coronary blockage was carried out at zero time of the graph and reperfusion was executed 5 min after the blockage. Upper trace of Fig. 3 obtained in untreated rats illustrates the normal sinus rhythm occurring during the initial 5 min of the experiment following the induced coronary blockage. Immediately after reperfusion was induced (the lower trace of Fig. 3) the occurrence of ventricular tachycardia and ventricular fibrillation was registered by the electrocardiogram

**Fig. 4:**
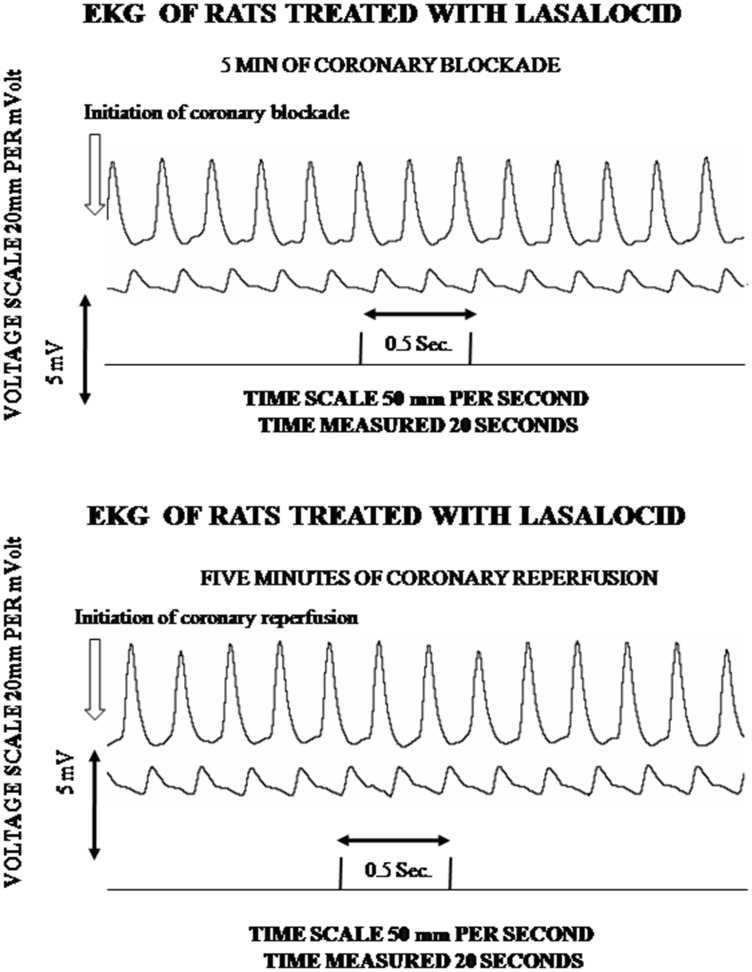
Effect of coronary blockage and reperfusion in the electrocardiogram of 5 rats which received 100 µl of ethanol-dimethyl formamide 3:1, containing 10 µM lasalocid. Lasalocid was injected intravenously in the femoral vein, 5 min before the blockage reperfusion. Coronary blockage was carried out at zero time of the graph and reperfusion was executed 5 more minutes after the blockage. Neither the coronary blockage (upper trace of Fig. 4) nor the subsequent coronary reperfusion (lower trace of Fig. 4) shows any detectable quantitative or qualitative modification in the electrocardiographic variations measured. It is also important to mention that the voltage scale of 20 mm per millivolt in *X* axes, as well as the time scale of 50 mm per second from the *Y* axes, were same before and after coronary blockade and reperfusion in Figs. 3 and 4

Figures 3 and 4 amplify the scope of the above statement by demonstrating that lasalocid fully protected and maintained stable and normal the electrical properties of the rat heart from the injury caused both by the blockage as well as by the reperfusion of the coronary artery. The upper trace of Fig. 3 illustrates that in the (*n*) = 20 untreated rats, the electrocardiographic trace was expressed with normal sinus rhythm during the initial 5 min of the experiment. However, when reperfusion was induced (lower trace of Fig. 3), an immediate occurrence of ventricular tachycardia and ventricular fibrillation, characteristic of reperfusion, was registered by the electrocardiogram, same which remained fully manifested and disordered, along the 5–15-min duration of the experiment, preceding the death of approximately 40% of the experimental subjects.

Strikingly, when the (*n*) = 20 experimental animals received 10 µM lasalocid, from 5 to 30 min before the start of the experiment, neither the coronary blockage (upper traces of Fig. 4), nor the subsequent coronary reperfusion (lower traces of Fig. 4), produced detectable quantitative or qualitative modifications in the electrocardiographic variations measured. This outstanding capability of lasalocid to maintain normal and unaltered the electrical heart performance was maintained practically normal in all experimental animals by the ionophore. The above evidence, which unequivocally shows normal electrocardiographic tracings, mediated or imposed by lasalocid, completely impeding the occurrence of ventricular tachycardia and fibrillation, was a valid reason to not further analyze the normal electrocardiograms through complementary standardized procedures, such as the Lambeth conventions.

On the other hand, it is also important to mention that unclear and negative results were obtained (*n* = 20) with lasalocid, similar to those shown in Panel A of Fig. 1 and in the black-dotted line of Panel A of Fig. 2, and lower trace of Fig. 3, when the ionophore was intravenously injected 5 or more minutes after reperfusion was in full process of development as well as when the lasalocid was administered anywhere from 2 h to 45 or more minutes prior to the induction of the coronary blockage and reperfusion procedure.

After the above data were obtained, a central question emerged from our results: how much of the energy conserving molecular and cellular properties of cardiomyocytes are maintained under normal physiological conditions, before and after the possible cell damage is caused by anoxia-reperfusion? With or without lasalocid? Results shown in Table 1 address this question. Indeed, Table 1 shows independent experiments measuring mitochondrial oxidative phosphorylation and coupled mitochondrial oxygen uptake, with the pair glutamate plus malate or with β hydroxybutyrate as oxidizable substrates in heart mitochondria obtained from control and reperfused hearts, with or without lasalocid in the experimental conditions described in methods.

In close cause and effect relationship with data from Fig. 1, 2, 3, and 4 and Table 1 shows that lasalocid completely prevents the abatement of mitochondrial oxidative phosphorylation and decay of net oxygen uptake induced by myocardial anoxia and cardiomyocyte damage, as caused by coronary reperfusion. In control hearts not subjected to reperfusion, mitochondrial oxidative phosphorylation P:0 ratios of 2.8 ± 0.12 were obtained with the pair glutamate plus malate, and P:0 ratios of 1.8 ± 0.15 were obtained with β hydroxybutyrate as oxidizable substrate. After reperfusion, in untreated hearts, not receiving lasalocid, P:0 ratios with glutamate plus malate, substantially decreased 65%, from of 2.8 ± 0.12 to 1.6 ± 0.03, as manifestation of cellular damage. Likewise, with β hydroxybutyrate as oxidizable substrate, the coupling of phosphorylation to electron transport also decreased more than 60% from the control values of 1.8 ± 0.15 to 0.62 ± 0.11.

Conversely, in reperfused hearts that received 10 µM lasalocid, 30 min before the blockage reperfusion process, lasalocid totally prevented the cellular or mitochondrial damage caused in the coupled multienzymatic process of electron transport and oxidative phosphorylation by anoxia reperfusion. Evidence for this is provided by the fact that, in reperfused hearts, with Lasalocid, P:0 ratios of 2.5 ± 0.12, were obtained with the pair glutamate plus malate. Against the diminished value of 1.6 ± 0.3 obtained without lasalocid and very similar to the control values of 2.8 ± 0.12. With an almost identical protective effect of lasalocid, obtained with β hydroxybutyrate, where lasalocid maintains practically normal the obtained P:0 ratios of 1.65 ± 0.18, well above the diminished value of 0.62 ± 0.11 found in reperfused rats without lasalocid, as compared with the normal control value of 1.8 ± 0.15.

Almost identical results to those observed in oxidative phosphorylation were also obtained when mitochondrial oxygen uptake was measured with both oxidizable substrates, with or without lasalocid. Emphasizing that all data obtained in the differences of oxygen uptake described below were statistically significant (*p* < 0.001). With the pair glutamate plus malate as oxidizable substrates, a ΔQO_2_ (*N*) of 242 microliters of O_2_ per mg of protein were consumed by mitochondria, from control non-reperfused hearts, in the absence of lasalocid. Also, reperfusion of the heart, without lasalocid with this substrate pair, abated 66% O_2_ uptake, to a value of 160 micro liters, whereas the presence of lasalocid in reperfused hearts, with glutamate plus malate, restores and elevates net oxygen uptake up, to 238 micro liters, very close, to the control value of 242. Almost identical comparative results, where lasalocid restores the loss of next oxygen uptake caused by myocardial damage provoked by reperfusion, were also obtained, when β hydroxybutyrate was used as oxidizable substrate. Where a ΔQO_2_ (N) value of 168 microliters of Oxygen were consumed in control non reperfused hearts, against a reduced value of 76 obtained in reperfused hearts without lasalocid and, up to 154 microliters in reperfused hearts with lasalocid. Very close also to the ΔQO_2_ (N) control value of 168μ liters of O_2_ obtained in mitochondria from controlled hearts oxidizing β hydroxybutyrate. Despite the significance of the above data, it is considered of value to further investigate mitochondrial protection function of lasalocid in live cardiomyocytes using gold standard assay for measuring mitochondrial respiration such as seahorse assay.

## Discussion

The results of this work indicate that the use of lasalocid, when intravenously administered from 5 to 30 minutes before the blockage and reperfusion of coronary artery in laboratory rats, immediately, totally and simultaneously interrupts ventricular tachycardia and fibrillation, blood pressure decay, cardiac frequency increase and abatement of mitochondrial oxidative phosphorylation, as well as decreases the mitochondrial oxygen uptake caused by coronary anoxia and reperfusion.

The molecular and cellular mechanism responsible for this sequence of events very likely involves the unique capability of the mobile ionophore to translocate Ca^2+^ by forming stereospecific complexes with the divalent cation, [5–7] as well as by transporting dopamine and norepinephrine through an exchange diffusion mechanism [10–13] occurring, across mitochondrial, cardiac cell membranes and through bimolecular lipid artificial membranes. This, in turn, very likely impedes the Ca^2+^ induced mitochondrial dysfunction occurring as an early event of the myocardial damage caused by anoxia-reperfusion [21] inducing the redistribution of intracellular calcium organelle pools, for example, the release of stored or bound calcium by mitochondria or sarcoplasmic reticulum, as suggested by Brooks et al. [14] thereby interrupting the apparent cytotoxic myocardial cell injury caused by the Ca^2+^ overload [14–16].

In support of above statement indicating a primary synergic role of calcium, as well as the catecholamines, dopamine and norepinephrine in cardiac function, there are data by Gelles [22] showing that lasalocid shortened the electric action potential and hyperpolarized the cardiac Purkinje cell fibers. Likewise, it has been demonstrated that lasalocid, in isolated normal hamster heart, generated an elevation of nitric oxide synthesis and the entry of calcium in the coronary vascular endothelium, thereby provoking an increase in the perfusion of coronary flux, when coronary blood flow was low [23].

Furthermore, in support of a key role of Ca^2+^, as well as the catecholamines norepinephrine and dopamine in the control of the molecular and cellular events leading to cardiac cell contractility, it has been found, in Sino auricular voltage clamp preparations from rabbit heart, that lasalocid elevated the length and strength of the contraction cardiac cycle at the same time that it increased calcium concentration in Purkinje cell fibers [24].

Additionally, a striking correlation [25] has been shown between extracellular calcium and the positive inotropism of cardiac muscle fibers induced by lasalocid. Likewise, it has also been demonstrated that at concentrations of 5 × 10^− 6^ M and 10^− 5^M, lasalocid elevated the contractile force of the ventricular muscle associated to high external concentration of potassium [26].

There is also unambiguous evidence by Pascual et al. [12] showing that the catecholamines dopamine and norepinephrine, as well as lasalocid, also participate in the contraction cardiac cycle and in the contractile inotropic properties of ventricular muscle through a Ca^2+^ independent process [27]. Preceded by findings showing that the Ca^2+^ ionophore also liberates dopamine from synaptosomal membranes from rat heart [13] and from isolated heart chromaffin vesicles [11], where Holz [10] showed that lasalocid also transports the neurotransmitters norepinephrine and dopamine, through artificial bimolecular lipid membranes. The above, clearly demonstrating that lasalocid, by itself, is also capable of specifically translocating the indicated neurotransmitters through cardiac cell membranes, thereby, equating its capability to specifically transport Ca^2+^ and no other mono, di- or trivalent cations across bimolecular artificial lipid membranes [6, 7].

The almost identical quantitative correlation existing between the dose–response requirements of lasalocid to prevent and restore myocardial damage caused by anoxia reperfusion (Panels A and B of Fig. 2), as well as to transport calcium, dopamine and norepinephrine, through biological [5] and bimolecular lipid membranes [6, 10] also indicates a possible common molecular mechanism governing such processes.

It is also of interest to mention that a number of chemicals have been introduced in the literature to reduce myocardial injury after reperfusion. Some of these substances have shown to be highly toxic, such as diazoxide and pinacidil, captopril, and cyclosporine [28] as well as, ryanodine [29], whereby others such as octylguanidine [19] or verapamil [30] look more promising. However, with the exception of verapamil [30], none has proven to exert beneficial effects as antibiotic, in chronic and acute diseases, also increasing weight and feed efficiency in long-term studies, such as lasalocid, [2–4]. Although most evidence shows that lasalocid does not cause undesirable effects in different animal species, at very high doses in long-term studies [1–4], it should be stressed that it has very specific cardio-protective effects, as shown in this manuscript, which occur at very low concentrations, in an acute, short and critical period of no more than 30 min, preceding myocardial damage and only in one single I.V. administration. This information does not preclude the possibility that lasalocid exerts its protective myocardial function also in long-term ischemia damage. For this purpose, TAC model and LAD ligation model could be of value. Furthermore, as mentioned before, it would be extremely useful to further investigate the mitochondrial protection function of the lasalocid in live cardiomyocytes, using gold standard assay for measuring mitochondrial respiration such as Seahorse assay.

## Conclusions

In conditions of near and imminent myocardial anoxic damage caused by coronary blockage and reperfusion, lasalocid appears to be one of the very few drugs in the heart pharmacopeia, in very specific condition, in a very short period of time and in an individual and low dose, that completely protects and maintains normal the integral functional properties of cardiomyocytes. In such conditions, the ionophore completely prevents blood pressure decay, cardiac frequency increase and ventricular tachycardia and fibrillation provoked by coronary reperfusion (Fig. 1, 2, 3, 4) maintaining fully functional the capability of the cardiac cells to couple mitochondrial electron transport to ATP synthesis, with β hydroxybutyrate and glutamate plus malate (Table 1). Oxidizable substrates that, as shown previously by our laboratory, are unaffected in their membrane translocation process by lasalocid [5] or nigericin [20], its molecular analog. This information not only strongly supports the validity of this latter conclusion, but also provides self-sufficient molecular and cellular evidence indicating the complete functional integrity of the cardiomyocyte in conditions of full prevention of myocardial damage, thereby rendering unnecessary any additional means to search for non-existent ventricular lesions.

Lasalocid appears to have significant limitations to impede the progress of an evolving heart lesion, if administered more than 1 h before or during or after a coronary blockage has already occurred. However, if administered during the period of 30 min or less before the onset of a possible ischemic myocardial damaging episode, lasalocid, undoubtedly and completely detains an anticipated ischemic heart lesion. Therefore, the present findings constitute relevant scientific evidence to stimulate the development of early warning technologies and electronic devices for personal use, such as intelligent watches, to detect and monitor with precision the early, simultaneous and parallel cardiac alterations of mechano-chemical, electrical, metabolic and hydraulic nature, preceding heart failure, to allow lasalocid, and other drugs of similar nature, to immediately detain an incoming ischemic myocardial lesion.
